# Delineating the Central Anatolia Transition Zone (CATZ): Constraints from Integrated Geodetic (GNSS/InSAR) and Seismic Data

**DOI:** 10.3390/s26020505

**Published:** 2026-01-12

**Authors:** Şenol Hakan Kutoğlu, Elif Akgün, Mustafa Softa

**Affiliations:** 1 Department of Geomatics Engineering, Faculty of Engineering, Zonguldak Bülent Ecevit University, Zonguldak 67100, Türkiye; shakan.kutoglu@beun.edu.tr; 2 Department of Geological Engineering, Fırat University, Elazığ 23200, Türkiye; 3 Department of Geological Engineering, Dokuz Eylül University, İzmir 35390, Türkiye; mustafa.softa@deu.edu.tr

**Keywords:** InSAR analysis, transition zone, lithospheric tearing, central Anatolia

## Abstract

Understanding how strain is transferred across the interior of tectonic plates is fundamental to quantifying lithospheric deformation. The Central Anatolia Transition Zone (CATZ), situated between the North and East Anatolian fault systems, provides a unique natural laboratory for investigating how continental deformation evolves from localized faulting to distributed shear. In this study, we integrate InSAR analysis with Global Navigation Satellite System (GNSS) velocity data, and stress tensor inversion with supporting gravity and seismic datasets to characterize the geometry, kinematics, and geodynamic significance of the CATZ. The combined geodetic and geophysical observations reveal that the CATZ is a persistent, left-lateral deformation corridor (i.e., elongated zone of Earth’s crust that accommodates movement where the landmass on the opposite side of a fault system moves to the left relative to an observer) accommodating ~4 mm/yr of shear between the oppositely moving eastern and western sectors of the Anatolian Plate. Spatial coherence among LiCSAR-derived shear patterns, GNSS velocity gradients, and regional stress-field rotations defines the CATZ as a crustal- to lithospheric-scale transition zone linking the strike-slip domains of central Anatolia with the subduction zones of the Hellenic and Cyprus arcs. Stress inversion analyses delineate four subzones with systematic kinematic transitions: compressional regimes in the north, extensional fields in the central domain, and complex compressional–transtensional deformation toward the south. The CATZ coincides with zones of variable Moho depth, crustal thickness, and inferred lithospheric tearing within the retreating African slab, indicating a deep-seated origin. Its S-shaped curvature and long-term evolution since the late Miocene reflect progressive coupling between upper-crustal faulting and deeper lithospheric reorganization. Recognition of the CATZ as a lithospheric-scale transition zone, rather than a discrete active fault, refines the current understanding of Anatolia’s kinematic framework. This study demonstrates the capability of integrated satellite geodesy and stress modeling to resolve diffuse intra-plate deformation, offering a transferable approach for delineating similar transition zones in other continental regions.

## 1. Introduction

Anatolia represents one of the most actively deforming continental regions on Earth, where the interactions of major lithospheric plates have shaped a complex and evolving tectonic framework. The Anatolian Plate—encompassing most of modern Türkiye—occupies a critical geodynamic position at the convergence of the Eurasian, African, and Arabian plates. The continuous northward motion of the African and Arabian plates imposes a compressional regime that drives the Anatolian Plate westward, away from the collisional front and toward the relatively stable Eurasian margin. This westward “tectonic escape” mechanism, governed by the wedge-shaped geometry of the Anatolian block, has been well established through decades of geological and geophysical research [1,2,3,4,5,6].

The westward extrusion of the Anatolian Plate is primarily accommodated by two major strike-slip fault systems: the North Anatolian Fault Zone (NAFZ) and the East Anatolian Fault System (EAFS) [3,4]. These transform boundaries delineate the principal deformation zones that have produced most of the region’s large historical and instrumental earthquakes. The 1939 Erzincan earthquake (Mw 7.9) on the NAFZ and the 2023 Kahramanmaraş earthquakes (Mw 7.8 and Mw 7.6) along the EAFS exemplify the ongoing seismic activity driven by plate convergence [7,8]. In contrast, western Anatolia experiences crustal extension, as demonstrated by the 2020 Samos earthquake (Mw 6.9) within the İzmir–Balıkesir Transfer Zone [9]. Collectively, these events highlight the elastic rebound process operating within a westward-drifting Anatolian microplate bounded by converging major plates.

While the westward motion of Anatolia is well constrained, the persistence of northward compression implies the existence of transitional domains that accommodate residual deformation and potentially mark zones of microplate interaction. Characterizing these zones is critical for understanding how strain is transferred between collisional and extensional regimes, especially across Central Anatolia, where the boundaries of deformation remain poorly defined.

Traditional geodetic approaches—particularly GNSS—have significantly advanced our understanding of Anatolia’s kinematics since the 1990s [10,11,12,13,14]. However, the limited station density and spatial resolution of GNSS networks hinder the detection of localized or slowly deforming structures. Recent studies integrating machine learning and GNSS velocity fields suggest a gradual transition from collision-dominated deformation in the east to slab-rollback-driven extension in the west [15,16,17,18,19]. Yet, the geometry, kinematics, and surface expression of this transitional zone remain unresolved, largely due to the absence of systematic InSAR-based investigations.

The advent of Interferometric Synthetic Aperture Radar (InSAR) has revolutionized the ability to monitor crustal deformation by providing continuous, high-resolution measurements of ground displacement over broad areas. The recent LICSAR dataset [20], which refers to the scientific data products generated by the Looking into Continents from Space with Synthetic Aperture Radar (LiCSAR) system, offers a comprehensive, countrywide InSAR-based deformation model for Türkiye, enabling consistent evaluation of subtle kinematic variations that may not be captured by point-based geodetic observations.

Over the past three decades, geodetic observations have played a central role in quantifying the kinematics of the Anatolian Plate and its interaction with surrounding plates. Early Global Navigation Satellite System (GNSS) studies provided the first robust constraints on plate motions and interseismic strain accumulation across the Eastern Mediterranean and Anatolia, establishing the westward extrusion of Anatolia and delineating major deformation zones associated with the North and East Anatolian fault systems [5,10,11,12,13,14]. Subsequent dense GNSS networks further refined the regional velocity field, revealing distributed strain, block-like behavior, and along-strike variations in deformation rates [15,16,17]. Recent advances, including machine-learning-based analyses of GNSS velocities, have emphasized gradual kinematic transitions from collision-dominated deformation in eastern Anatolia to extension-related processes in the west, suggesting the presence of transitional deformation domains rather than sharp tectonic boundaries [18,19].

Despite these advances, GNSS-only approaches remain spatially limited in resolving localized or diffuse deformation due to station spacing. The increasing availability of Interferometric Synthetic Aperture Radar (InSAR) data has therefore motivated integrated InSAR–GNSS methodologies, in which GNSS velocities provide a stable reference frame and long-wavelength constraints, while InSAR captures high-resolution spatial variations in surface deformation. The development of automated, large-scale InSAR processing systems such as LiCSAR has enabled consistent, countrywide deformation mapping across Türkiye, allowing subtle kinematic gradients and previously unresolved deformation zones to be identified [20]. Recent studies increasingly recognize that combining GNSS and InSAR velocity fields is essential for characterizing transitional tectonic zones, strain partitioning, and deformation heterogeneity within Anatolia, particularly in regions where deformation is not fully accommodated by major fault systems alone [16,17,18].

Building on these advances, this study integrates multi-temporal InSAR observations with existing GNSS velocity fields to (i) identify and quantify the spatial distribution of residual deformation within Central Anatolia, (ii) evaluate the presence and geodynamic role of transitional tectonic zones between the EAFS and the extensional domain of western Anatolia, and (iii) refine the current understanding of Anatolia’s three-dimensional surface deformation field. The results provide new insights into the plate-interaction dynamics that govern Anatolia’s active tectonics and contribute to improved regional seismic hazard assessment.

## 2. Seismotectonic Setting

**Figure 1:**
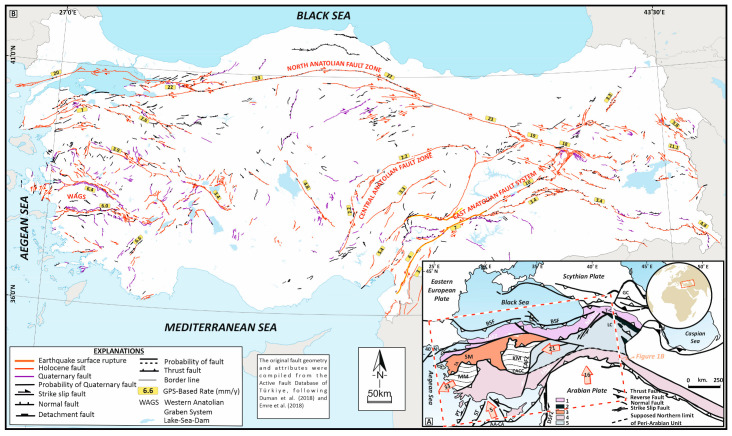
(**A**) Simplified geologic and tectonic framework of the Anatolia–Caucasus region (modified after [23,24]). Major deformation zones include the NAFZ—North Anatolian Fault Zone, CAFZ—Central Anatolian Fault Zone, EAFS—East Anatolian Fault System, and NEAFZ—Northeast Anatolian Fault Zone. Additional features: BSF—Black Sea Fault; DSFZ—Dead Sea Fault Zone; PT—Pliny Trench; ST—Strabo Trench; AA–CP—Aegean–Cyprus Arc; GC—Greater Caucasus; LC—Lesser Caucasus; KM—Kırşehir Massif; MM—Menderes Massif; SM—Sakarya Massif; and CACC—Central Anatolian Crystalline Complex. Light orange arrows denote plate motion vectors (mm/yr) relative to stable Eurasia [25]. (**B**) Seismotectonic map of Türkiye (modified after [26,27]), illustrating the distribution of seismicity and active fault systems defining the present-day deformation field of Anatolia.

Türkiye occupies a key geodynamic position within the Alpine–Himalayan orogenic belt, where remnants of the Gondwana- and Eurasia-derived terranes have amalgamated through successive episodes of subduction, collision, and accretion from the Paleozoic to the present. The interaction of the Eurasian, Arabian, and African plates has produced a complex lithospheric mosaic that underpins the present-day neotectonic framework of Anatolia [4], (Figure 1A). The resultant tectonic configuration is expressed in a range of crustal deformation styles—from continental collision in the east to crustal extension in the west—leading to pronounced lateral variations in seismogenic depth and crustal structure.

Since the Miocene, the Anatolian Plate has undergone westward and southwestward extrusion, driven by oblique convergence between the Arabian and Eurasian plates and by subduction rollback along the Aegean–Cyprus Arc (AA–CA) [21,22]. The active deformation of Türkiye is therefore governed by multiple processes, including continental collision, subduction, crustal extension, and strike-slip faulting, which together define the modern seismotectonic regime.

### 2.1. Major Active Tectonic Zones

The fundamental tectonic structures accommodating present-day deformation include the North Anatolian Fault Zone (NAFZ), East Anatolian Fault System (EAFS), Central Anatolian Fault Zone (CAFZ), Dead Sea Fault Zone (DSFZ), Bitlis Suture Zone (BSZ), Western Anatolian Graben System (WAGS), and the Aegean–Cyprus Arc (AA–CA), (Figure 1B). These inherited plate-boundary systems represent the most seismically active zones in Türkiye [26,27,28,29]. The destructive earthquake sequence between 2020 and 2023, spanning the EAFS, NAFZ, and WAGS, underscores the high seismic potential of these fault systems and their complex segment interactions.

The NAFZ is one of the world’s most prominent intra-continental right-lateral strike-slip systems, extending ~1400 km from the Karlıova triple junction to the northern Aegean Sea [4,5,27,30]. Composed of at least 18 major fault segments, the NAFZ displays east–west variations in fault geometry and strain width—narrow in the east and bifurcated toward the west. Geological and geodetic observations indicate a slip rate of 5–25 mm/yr [25,31,32]. To its northeast, the Northeastern Anatolian Fault Zone (NEAFZ) and the Black Sea Fault (BSF) form an integrated dextral–thrust system that accommodates part of the regional strain [33,34,35,36].

The EAFS forms the principal left-lateral fault boundary between the Arabian Plate and the Anatolian microplate. Comprising 16 segments, it accommodates 4–31 mm/yr of sinistral motion [25,37,38]. The deformation zone widens toward the southwest as the slip rate decreases, reflecting distributed strain partitioning between parallel strands [39]. The BSZ to the south marks the zone of continental collision, characterized by thrust and strike-slip structures that evolved with the initiation of the EAFZ in the late Pliocene [3,29].

The CAFZ represents a major intra-plate sinistral strike-slip system within Central Anatolia, reactivated during the neotectonic period [26,40]. Its branches—including the Ecemiş and Deliler faults—display mixed strike-slip and normal motion, with GNSS constrained slip rates of 2–5 mm/yr [25,41,42]. These faults accommodate distributed strain between the NAFZ and EAFZ and likely play a role in the progressive westward motion of Anatolia.

The WAGS delineates the western extensional province of Türkiye, characterized by active normal faulting and rapid crustal thinning at rates up to 40 mm/yr [29,43]. The Gediz, Küçük Menderes, and Büyük Menderes grabens are the principal basins reflecting slab rollback and back-arc extension associated with the Hellenic subduction system [22,44]. The zone transitions eastward into strike-slip deformation linking with the southern branch of the NAFZ [45,46,47,48,49].

To the south, the Aegean–Cyprus Arc (AA–CA) forms the active subduction front between the African and Anatolian plates. The Pliny and Strabo trenches represent the surface expression of this subduction zone, which generates intermediate-depth seismicity associated with the northward subduction of the African lithosphere [50,51].

### 2.2. Central Anatolia: The Transitional Domain

Central Anatolia lies between the compressional regime of eastern Türkiye and the extensional domain of the Aegean region, representing a key tectonic transition zone [4,52]. The area is bounded by the NAFZ to the north and the EAFS to the southeast, and contains several secondary fault systems—including the Tuz Gölü, Ecemiş, and Ezinepazarı faults—that accommodate intraplate deformation [5,53,54]. These “germanotype” faults [55] display mixed kinematics, transitioning from compressional in the east to extensional in the west, reflecting the complex strain transfer between major plate boundaries.

Geodetic studies [13,25] indicate that Central Anatolia moves southwestward as a relatively rigid block within a diffuse shear zone, sometimes referred to as the “Anatolian Diagonal” [56]. This wide left-lateral shear zone, composed of second-order faults, plays a critical role in the internal deformation of Anatolia and in mediating strain between contrasting tectonic domains.

### 2.3. Geodynamic Evolution

The long-term evolution of Central Anatolia has been shaped by Mesozoic–Cenozoic subduction and orogenic processes along the İzmir–Ankara–Erzincan Suture Zone [3,57,58]. Continental and oceanic nappes accumulated in east–west-trending belts, while subduction rollback and slab tearing beneath the Cyprus trench drove Late Cenozoic uplift and volcanism [59,60]. Seismic tomography and full-waveform models [61,62] support slab fragmentation and horizontal tearing of the subducted African lithosphere beneath Central Anatolia, providing a plausible explanation for the region’s complex deformation and widespread volcanism.

### 2.4. Implications for Remote Sensing and Geodesy

This multi-scale tectonic framework results in spatially variable deformation patterns that are ideal for observation using modern remote sensing techniques. InSAR and GNSS data jointly enable quantification of both distributed and localized strain, offering new insights into the partitioning of deformation across the transitional domains of Central Anatolia. Understanding these spatial variations is crucial not only for refining regional kinematic models but also for improving seismic hazard assessments in one of the most geodynamically active regions of the Mediterranean.

## 3. Methodology

The integration of InSAR, GNSS, and stress inversion datasets provides a multi-dimensional framework for investigating both the kinematic and dynamic characteristics of crustal deformation in Anatolia. InSAR captures fine-scale, spatially continuous surface displacements; GNSS constrains absolute motion and long-wavelength strain; and focal mechanism inversions elucidate the active stress field driving faulting. By correlating spatial gradients in the LiCSAR-derived velocity field with SHmax orientations from stress inversion, we identify zones of strain concentration and potential kinematic transitions between the strike-slip and extensional domains of Anatolia. This integrated geodetic–seismological approach enables improved quantification of fault interaction, strain partitioning, and seismic hazard across one of Earth’s most actively deforming continental regions.

### 3.1. LiCSAR-Derived Velocity Fields

LiCSAR is an automated interferometric processing framework developed by the COMET (Centre for the Observation and Modelling of Earthquakes, Volcanoes, and Tectonics) consortium to systematically generate Sentinel-1 interferometric products [20]. Sentinel-1 data have been acquired on every 12-day revisit since Sentinel-1 satellite mission were launched in October 2014. Sentinel-1 data are acquired in both ascending and descending orbits. After InSAR processing of the SAR acquisitions from these two viewing geometries, the resulting line-of-sight (LOS) displacements are jointly exploited to derive the horizontal and vertical displacement components.

**Figure 2:**
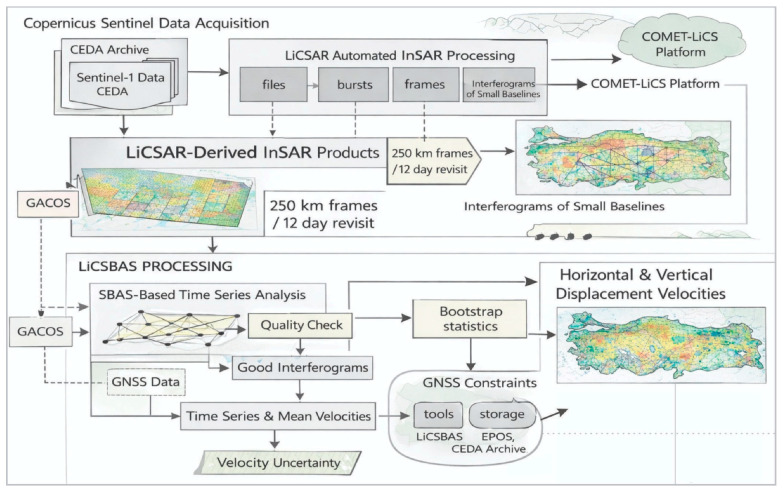
Summary of processing schemes supporting the integrated LiCSAR–LiCSBAS InSAR analysis, revised from [20,64].

Sentinel-1 data processed within the LiCSAR automated processing framework are provided to users via the COMET-LiCSAR platform as open-access, downloadable products at the ~250 × 250 km frame scale (https://comet.nerc.ac.uk/comet-lics-portal/, accessed on 30 July 2024). To derive InSAR time series from LiCSAR frame products, the Small Baseline Subset (SBAS) technique is utilized. In SBAS InSAR, interferograms are generated from image pairs with small temporal and/or spatial (perpendicular) baselines to construct a high-coherence interferometric network, from which the displacement time series and mean velocity are estimated through inversion. LiCSBAS v2.0 is an open-source SAR interferometry time-series analysis package developed by COMET based on the SBAS technique. Using this software (LiCSBAS v2.0), InSAR displacement time series are derived from LiCSAR products acquired in both ascending and descending orbits (Figure 2).

SBAS processing yields line-of-sight (LOS) displacements from both ascending and descending geometries. They are then decomposed into horizontal and vertical components using the following equation.

$$v_{LOs}=\left[sin\theta -sin\theta sin\alpha -cos\theta \right]\left[\begin{array}{c} v_{E}\\ v_{N}\\ v_{U} \end{array}\right]$$

where $v_{LOs}$ is the LOS velocity, θ is the local radar incidence angle, α is the azimuth of the satellite heading vector, and ${\left[v_{E}\;v_{N}\;v_{U}\right]}^{T}\;$ is the vector of displacements in directions of East, North and Up, respectively.

Sentinel-1 flies on a near north–south (quasi-polar) orbit and its radar is right-looking. Consequently, it is largely insensitive to north–south (N–S) displacement. To recover the full three-dimensional velocity field in the above equation, it is therefore common to incorporate GNSS-derived velocity constraints.

In this study, we used LiCSAR/LiCSBAS-derived InSAR data over Türkiye for the period 2015–2019. This dataset was selected because it has been validated and widely adopted in the literature (see [20,63,64]). The data cover 14 overlapping Sentinel-1 tracks, comprising 7 ascending and 7 descending acquisition geometries. In LiCSAR processing, 40 frames of approximately 250 × 250 km were defined, and for each frame an interferogram network comprising ~600–800 interferograms generated from ~200 acquisitions was constructed. The interferograms were multilooked to yield ~80 × 80 m ground pixels (resampled to ~100 m spacing during geocoding), and the interferometric phase was unwrapped using SNAPHU. Atmospheric contributions were partially mitigated using GACOS, reducing the interferogram phase standard deviation by ~20–30% on average (Figure 2).

To generate InSAR LOS displacement time series and velocities over Anatolia, LiCSAR data were processed using LiCSBAS v2.0 software. The data were further downsampled by a factor of 10 to a pixel size of ~1 km, which is sufficient for large-scale tectonic applications. Velocity uncertainty was estimated using the percentile bootstrap method, and pixels were masked based on several noise indices. Finally, the root-mean-square (RMS) difference in the resulting velocities was approximately 2 mm/yr.

After LiCSAR/LiCSBAS processing, each frame has its own independent reference point for velocity estimation. Therefore, the derived LOS velocities were transformed into a Eurasia-fixed reference frame using regional GNSS velocity data. The ascending and descending LOS velocities obtained in this way were then decomposed into their three-dimensional components using the above equation, incorporating the GNSS velocity field.

GNSS data were compiled from long-term campaigns and continuous stations operated by the Turkish National Permanent GNSS Network (TUSAGA-Active) and from published velocity solutions [16,25,65]. All GNSS velocities were expressed in the ITRF2014 reference frame and subsequently transformed into a Eurasia-fixed frame using stable reference sites located in the northern part of the plate, following the methodology of [13]. The station spacing of the TUSAGA-Aktif network is on the order of 70–80 km. In the literature, differences between independently processed GNSS solutions and the published velocity estimates have been reported to be on the order of ~1–2 mm/yr in the horizontal components and a few mm/yr in the vertical component.

### 3.2. Stress Tensor Inversion Analysis

To characterize the active stress regime, we performed stress tensor inversion using earthquake focal mechanism data. The inversion estimates the principal stress orientations and stress ratio (R) by minimizing the angular deviation between observed and predicted slip directions under a uniform stress field assumption [66,67,68].

Focal mechanisms (Mw ≥ 3.8) were compiled from multiple authoritative sources, including the Disaster and Emergency Management Authority of Türkiye (AFAD), the Global Centroid Moment Tensor (GCMT) catalog, Harvard CMT (HRVD), and the International Seismological Centre (ISC), yielding a total of 79 high-quality events (Supplementary Table S1). Each mechanism defines two orthogonal nodal planes, one of which represents the true fault plane. The inversion assumes a homogeneous stress field in space and time and that fault slip occurs in the direction of maximum shear stress (τ), following the Wallace–Bott hypothesis [69,70].

Stress inversions were conducted using Win-Tensor v.5.9.2 software [67] employing the Rotational Optimization (F5) algorithm [71] to reject incompatible solutions and identify the most probable fault plane. The outputs include the orientations of the three principal stress axes (σ_1_ > σ_2_ > σ_3_), the stress ratio (R = (σ_2_ − σ_3_)/(σ_1_ − σ_3_)), and the azimuth of maximum horizontal compressive stress (SHmax). Focal mechanism types (normal, strike-slip, thrust) were classified using the [72] ternary diagram, linking seismological stress orientations with observed deformation patterns. Stress tensor inversion quality was assessed using the composite function of Delvaux and Sperner [67] within the World Stress Map (WSM) framework [68]. The inversion procedure was constrained such that only solutions with mean slip misfit angles ≤ 30° were retained for further analysis.

## 4. Result

### 4.1. LiCSAR-Derived Surface Deformation Patterns

**Figure 3:**
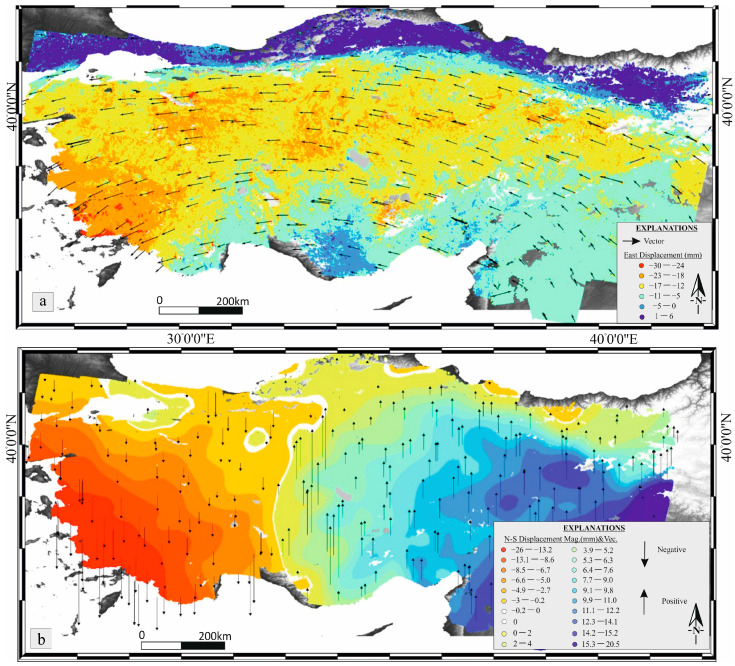
LICSAR-derived surface displacements at 1 km × 1 km resolution: (**a**) horizontal displacement and east–west component, and (**b**) north–south component, illustrating the spatial distribution and orientation of crustal deformation across the study area.

The LiCSAR-derived velocity fields illustrate the spatial distribution of deformation across Anatolia in three components: total horizontal displacement (Figure 3a), the east–west component (Figure 3a), and the north–south component (Figure 3b). The horizontal displacement field (Figure 3a) and the east–west component (Figure 3a) exhibit consistent patterns, both dominated by the westward extrusion of the Anatolian Plate relative to the Eurasian Plate. This movement is accommodated primarily along two major strike-slip fault systems: the North Anatolian Fault Zone (NAFZ) and the East Anatolian Fault System (EAFS).

In contrast, the north–south component (Figure 3b) reveals a distinct and complex deformation pattern that is not apparent in the other components. Eastern Anatolia shows northward motion at rates up to approximately 20 mm/yr, whereas western Anatolia displays southward motion reaching −25 mm/yr. Between these opposite displacement domains lies a continuous north–south-trending belt extending across central Anatolia. This belt exhibits coherent left-lateral motion, representing a transition zone here defined as the Central Anatolia Transition Zone (CATZ).

The CATZ accommodates an average left-lateral displacement rate of ~4 mm/yr. The NAFZ, acting as a northern mechanical boundary, imposes an S-shaped deflection on the CATZ trace, extending approximately 119 km in length. Assuming a slip rate of 20 mm/yr along the NAFZ, this bending geometry could have developed over roughly 5.9 million years. At the northern termination of the CATZ, near the Black Sea, the deformation produces an arc-shaped coastal morphology whose orthogonal width (~24 km) corresponds to a similar timescale of deformation (≈5.9 Myr) at the same velocity. These geometric and kinematic relations suggest that the CATZ has been a long-lived structural feature accommodating left-lateral shear across central Anatolia.

### 4.2. Stress Tensor Inversion Results

**Figure 4:**
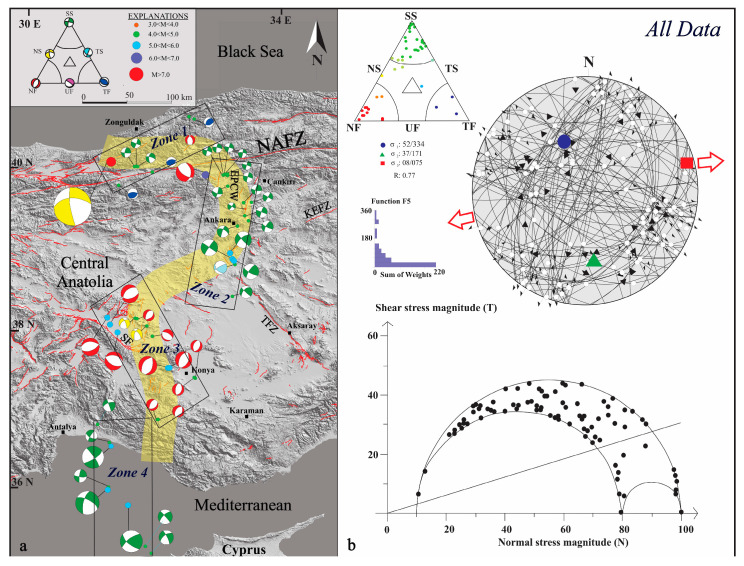
(**a**) Distribution of earthquake focal mechanisms along the Central Anatolia Transition Zone, compiled from AFAD and GCMT catalogs, overlaid on the active fault map [27]. (**b**) Stress inversion results derived from all earthquake data using Win-Tensor software [67,71]. Key structures: NAFZ—North Anatolian Fault Zone; TFZ—Tuz Gölü Fault Zone; KEFZ—Ezinepazarı Fault Zone; EPCW—Eldivan–Elmadağ Pinched Crustal Wedge; SF—Sultandağı Fault. The map illustrates the spatial variation in stress regime and kinematics across the transition zone. Colors of the data points within the Frohlich ternary diagram indicate the dominant faulting style inferred from focal mechanisms: blue—thrust faulting; green—strike-slip faulting; yellow—oblique strike-slip with a normal faulting component; and red to pink—normal faulting.

A total of 43 focal mechanism solutions were inverted to determine the regional stress field along the CATZ. The study area was subdivided into four structural zones (Zones 1–4) defined by geodetic and seismotectonic criteria (Figure 4a). The overall inversion indicates an extensional stress regime (R′ = 0.77 ± 0.17) characterized by a NNW–SSE-trending maximum horizontal stress axis (SHmax = N016° W ± 7.5°) and an ENE–WSW-trending minimum horizontal stress axis (SHmin). The resulting stress regime is transtensional, reflecting the coexistence of strike-slip and extensional deformation (Figure 4b and Table 1).

**Figure 5:**
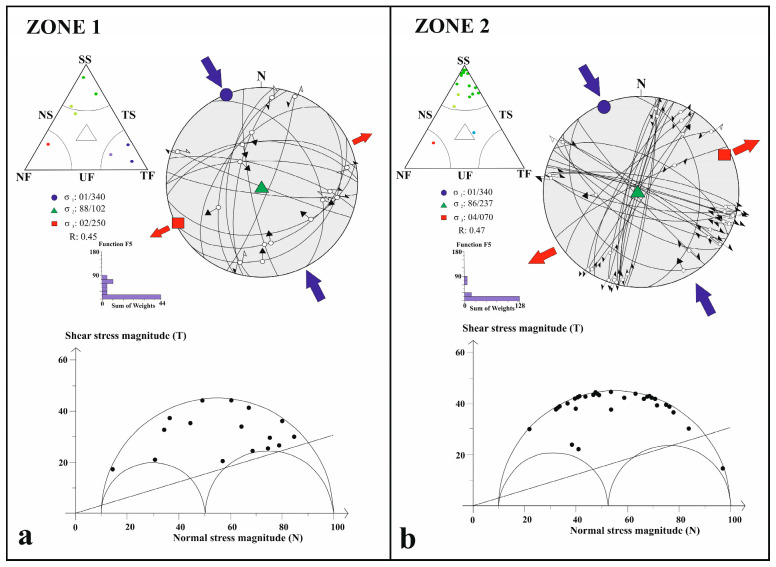
Stress inversion results derived from Zone 1 (**a**) and from Zone 2 (**b**) using Win-Tensor software [67,71], illustrating the orientation and style of the regional stress field across the study area. Blue arrows indicate the horizontal component of the maximum principal stress (σ_1_), whereas red arrows represent the horizontal component of the minimum principal stress (σ_3_). Colors of the data points within the Frohlich ternary diagram indicate the dominant faulting style inferred from focal mechanisms: blue—thrust faulting; green—strike-slip faulting; yellow—oblique strike-slip with a normal faulting component; and red to pink—normal faulting.

Zone 1 corresponds to the northernmost sector of the CATZ and coincides with the concave bend of the NAFZ. The stress inversion for this zone yields a strike-slip tensor (R′ = 1.55 ± 0.18) with SHmax oriented N021° W ± 9.3°, consistent with dextral strike-slip kinematics. The largest earthquake within this zone, the 1999 Düzce event (Mw 7.1, 18 km depth), produced a transtensional stress state (R′ = 1 ± 0.15; SHmax N054° W ± 16.1°), though the cumulative inversion indicates a dominant strike-slip regime (Figure 5a, Table 1). These results confirm that the NAF controls the northern termination of the CATZ through dextral shear, while localized transtension arises from fault curvature.

Zone 2 corresponds to the region influenced by the Eldivan–Elmadağ Pinched Crustal Wedge (EPCW), a transitional structure between compressional and extensional tectonic domains [54]. The inversion of the largest event (Mw 6.0, 15 km depth) yields an extensional stress tensor (R′ = 0.5 ± 0.10; SHmin N061° E ± 13.1°) indicating normal faulting along the wedge’s western margin. The cumulative inversion for all events gives a strike-slip tensor (R′ = 1.53 ± 0.4; SHmax N020° W ± 12.5°), reflecting a left-lateral strike-slip regime (Figure 5b, Table 1). These results indicate that Zone 2 acts as a structural transfer zone accommodating sinistral motion within a transtensional framework.

**Figure 6:**
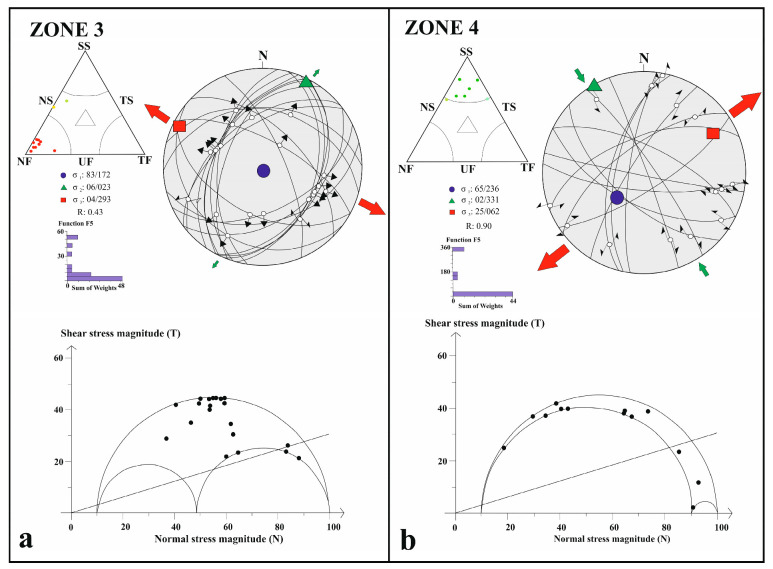
Stress inversion results for Zone 3 (**a**) and Zone 4 (**b**) obtained using Win-Tensor software [67,71], showing the orientation and style of the stress field across the transition zone. Green arrows indicate the horizontal component of the intermediate principal stress (σ_2_), whereas red arrows represent the horizontal component of the minimum principal stress (σ_3_). The colors of the data points within the Frohlich ternary diagram indicate the dominant faulting style inferred from focal mechanisms: blue—thrust faulting; green—strike-slip faulting; yellow—oblique strike-slip with a normal faulting component; and red to pink—normal faulting.

Zone 3, aligned with the NW–SE-trending Sultandağı Fault Zone, displays a stress field distinct from Zones 1 and 2. The 2002 Sultandağı earthquake (Mw 6.5, 15 km depth) provides an extensional stress tensor (R′ = 0.5 ± 0.10; SHmin N018° W ± 16.1°), consistent with normal faulting. Surface rupture data show up to 21 cm vertical offset, 15 cm horizontal extension, and 10 cm left-lateral slip [73]. The cumulative inversion (R′ = 0.43 ± 0.22; SHmax N022° E ± 19.5°) confirms a dominantly extensional regime with minor sinistral component (Figure 6a, Table 1). These findings indicate that the zone marks the southward transition from strike-slip to extensional deformation.

Zone 4 encompasses the southernmost segment, where the CATZ merges with the complex fault systems of the Eastern Mediterranean, particularly the Antalya–Kekova Fault Zone (AKFZ) and Biruni Fault Zone (BFZ). The inversion results yield a compressive stress tensor (R′ = 0.90 ± 0.17; SHmax N028° W ± 7.4°), reflecting a transtensional regime associated with subduction along the Hellenic–Cyprus arc (Figure 6b, Table 1). Despite local variations in slip sense—dextral or sinistral—the focal mechanisms indicate that deformation continuity extends from the central Anatolian interior into the Mediterranean domain.

## 5. Discussion

The selection of the Central Anatolia region for this study is motivated by its unique tectonic position between two of the most seismically and geodynamically active plate boundaries on Earth—the North Anatolian Fault Zone (NAFZ) to the north and the East Anatolian Fault System (EAFS) to the southeast. This region forms the internal domain of the Anatolian Plate, where strain transfer between these major strike-slip systems is far from straightforward. While the NAFZ and EAFZ accommodate the principal plate-boundary displacements through well-defined fault strands, the interior of Anatolia exhibits a more distributed deformation pattern, suggesting that the transfer of motion between these boundaries is mediated by a broad zone rather than a single, throughgoing fault. This complexity makes Central Anatolia an ideal natural laboratory for evaluating how lithospheric-scale deformation evolves away from discrete fault lines into diffuse, distributed zones of strain.

According to plate tectonic theory, lithospheric plates behave as rigid blocks moving over the asthenosphere at rates up to 10 cm/yr [74]. However, this idealization breaks down within continental interiors, where strain is rarely transmitted uniformly through the seismogenic crust. Instead, deformation becomes partitioned across multiple structures and manifests as distributed shear, folding, or ductile flow [75,76,77,78,79]. Geodetic observations from GNSS and InSAR datasets across central Anatolia reveal precisely such distributed strain, with velocity gradients (~4 mm/yr) incompatible with a single active fault model [25,42].

The spatial continuity, deformation style, and kinematic coherence of the Central Anatolia Transition Zone (CATZ) strongly support its interpretation as a tectonic transition zone rather than a discrete fault system. The 5.9 Myr evolution of its S-shaped geometry and northern arc structure coincides with the onset of late Miocene to Pliocene intracontinental deformation [40,80,81]. This temporal link emphasizes that the CATZ is not a recent or localized feature, but a long-lived structural domain that accommodates lithospheric-scale adjustment between contrasting deformation regimes of Anatolia.

Stress tensor inversions further confirm a systematic southward transition in tectonic regime—from dextral strike-slip along the NAFZ (Zone 1), through sinistral transtension in central Anatolia (Zones 2–3), to compression and transpression within the Mediterranean domain (Zone 4). This coherent pattern reflects the interplay of two dominant geodynamic mechanisms: (1) the westward extrusion of the Anatolian Plate driven by Arabia–Eurasia convergence, and (2) the southwestward rollback of the Hellenic–Cyprus subduction system. Their combined influence generates a rotational strain field where shear, extension, and compression coexist, defining central Anatolia as a zone of mechanical and kinematic transition.

The CATZ is better interpreted as a broad, left-lateral deformation corridor rather than a single, through-going crustal fault. In contrast to classical strike-slip fault zones that localize deformation along discrete structures and produce continuous surface ruptures, no continuous or well-defined surface fault trace can be mapped that accounts for the regional kinematics of the CATZ. This absence of a crustal-scale, localized fault suggests that deformation is not confined to a single brittle structure. Stress tensor inversions derived from earthquake focal mechanisms reveal a systematic southward transition in tectonic regime from Zone 1 to Zone 4. This progressive spatial variation in stress orientation and faulting style is inconsistent with slip on a single planar fault and instead indicates distributed strain across a wide region. Such behavior implies a gradual partitioning of deformation within the crust and uppermost mantle rather than sharp localization at the surface. This deformation pattern reflects a transition from predominantly brittle faulting in the upper crust to more ductile or semi-brittle behavior at depth, facilitated by rheological heterogeneity, inherited structural anisotropy, and lateral variations in crustal thickness across Central Anatolia. Consequently, the lack of a continuous surface rupture does not indicate tectonic quiescence; instead, it points to a diffuse mode of strain accommodation involving both the crust and upper mantle.

**Figure 7:**
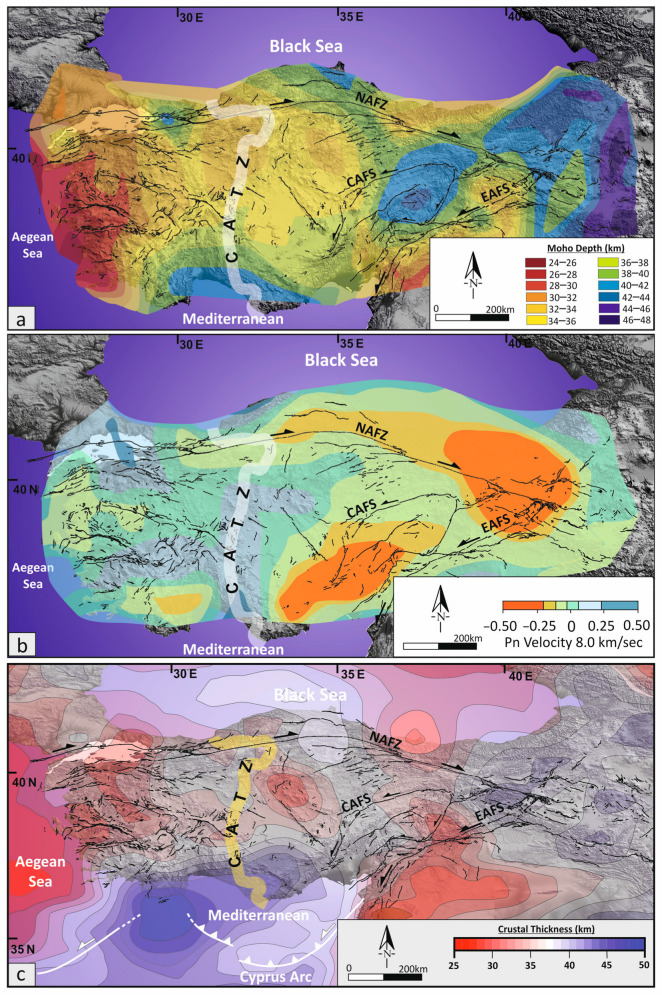
(**a**) Moho depth map of Turkey reprojected and simplified from [82], highlighting major crustal thickness variations across tectonic domains (**b**) Pn velocity anomalies and (**c**) crustal thickness across Turkey, illustrating variations in lithospheric structure reprojected and simplified from [83]. (**c**) Active fault map from [30], NAFZ: North Anatolian Fault Zone, EAFS: East Anatolian Fault System, CAFS: Central Anatolian Fault System, CATZ: Central Anatolian Transition Zone.

Consequently, the CATZ should be viewed as a lithospheric-scale transition zone, where plate-boundary forces, crustal rheology, and mantle dynamics interact to define the evolving deformation field of Anatolia. Integrating InSAR-derived strain fields with stress tensor analyses reveals that this zone not only accommodates ongoing intra-plate shear but also corresponds spatially with key crustal and mantle anomalies identified by independent geophysical observations. Three alternative, yet potentially complementary, geodynamic scenarios may explain the origin and persistence of the CATZ: (1) a lithospheric slab tear or detachment zone, (2) a crustal heterogeneity related to Moho depth variation, and (3) a localized crustal thickness transition and rheological boundary contrast (Figure 7).

1. Slab Tear or Lithospheric Tearing Associated with Plate Convergence: The first scenario interprets the CATZ as a surface expression of a deeper lithospheric tear developed during the complex convergence of the African, Arabian, and Eurasian plates. The geometry of the deformation belt, together with the north–south orientation of the LiCSAR-derived shear lineaments, coincides with the inferred northern termination of the subducted African lithosphere beneath central and southern Anatolia [18,62,84,85,86,87]. In this model, the differential rollback and tearing of the Cyprus–Hellenic slab have induced a localized upwelling and lateral extrusion of the overlying crust, giving rise to the observed shear-dominated strain field.

The consistency between the left-lateral motion observed in the CATZ and the southwestward motion of the Anatolian microplate supports this interpretation. Furthermore, the southward transition from strike-slip to transpressional regimes reflects the mechanical coupling between the upper crust and a deforming, partially detached lithospheric slab. The development of the S-shaped curvature and the inferred 5.9 Myr deformation timescale are compatible with progressive slab segmentation since the late Miocene, as previously documented along other retreating subduction margins [88]. Thus, the CATZ may mark the northern surface projection of a deep-seated tear propagating through the lower lithosphere.

2. Crustal Heterogeneity and Moho Depth Variation: An alternative explanation attributes the CATZ to crustal-scale rheological heterogeneity associated with lateral Moho depth variations across central Anatolia. Seismic and magnetotelluric profiles reveal pronounced crustal thinning from the Central Anatolian plateau (~40 km) toward the Mediterranean domain (~30 km) [82,89,90]. The observed InSAR deformation gradient (~4 mm/yr) may therefore reflect a mechanically weak transition between thick, rigid continental crust in the north and a thinned, ductile lithosphere in the south.

This interpretation aligns with the southward increase in extensional strain and with the spatial overlap between the CATZ and regions of reduced crustal seismic velocity. The stress tensor inversion, showing a systematic rotation from strike-slip to transtensional regimes, further indicates strain localization at the boundary between crustal domains of differing strength. The deformation observed by LiCSAR and GNSS thus represents a surface manifestation of this crustal transition, consistent with the hypothesis that the CATZ is mechanically controlled by Moho topography and lithospheric thickness contrasts rather than by active slab tearing alone.

3. Crustal Thickness Transition and Rheological Boundary: An alternative geophysical framework considers the CATZ as the surface expression of a major crustal thickness transition across Central Anatolia. Moho depth variations mapped by receiver-function and surface-wave analyses [83] show a sharp gradient between the thickened crust of Central Anatolia and the thinner crust toward the Tauride belt. This transition defines a mechanically heterogeneous zone in which contrasts in crustal strength and effective viscosity promote differential horizontal and vertical deformation. Such rheological contrasts are expected to strain partition across a wide region, enhancing diffuse shear along inherited structural fabrics rather than focusing deformation onto a single fault.

Within this framework, the left-lateral kinematics of the CATZ reflect deformation accommodated across a crustal-scale boundary governed by variations in crustal thickness and mechanical strength, rather than by a narrow lithospheric-scale discontinuity. The spatial correspondence between Moho depth gradients and the distributed deformation pattern identified in this study supports the interpretation that the CATZ marks a fundamental thermomechanical boundary within the Anatolian lithosphere.

Although each scenario provides a plausible contribution, the combined InSAR, GNSS, and stress-tensor results indicate that the CATZ results from the interaction of deep lithospheric processes and crustal-scale rheological heterogeneity. Variations in Moho depth capture the primary crustal control on strain distribution, while deeper mantle dynamics modulate the regional stress field. Together, these processes produce a distributed deformation regime that couple’s vertical crustal adjustment with horizontal shear partitioning.

This interpretation reinforces the view that the Anatolian Plate does not behave as a rigid microplate but undergoes significant internal deformation along zones of diffuse shear. The CATZ thus emerges as a key lithospheric boundary mediating strain transfer between strike-slip–dominated deformation in Central Anatolia and subduction-related processes along the Tauride margin.

Although the non-rigid behavior of the Anatolian Plate is evident from its complex tectono-geological setting and heterogeneous topography, these observations do not constrain present-day strain accommodation. Here we evaluate three alternatives, yet potentially complementary, mechanisms for the CATZ: a lithospheric slab tear, Moho depth–related crustal heterogeneity, and a localized crustal thickness transition forming a rheological boundary. Comparisons with InSAR-, GNSS-, and stress-tensor results show that diffuse shear within the CATZ reflects an actively maintained deformation regime controlled by current lithospheric structure, rather than passive inheritance from past orogenic processes.

## 6. Concluding Remarks and Conclusions

The integration of InSAR-derived deformation data, GNSS velocity fields, and stress tensor inversions provides new insight into the lithospheric deformation and dynamic evolution of central Anatolia. The findings clearly demonstrate that the Central Anatolia Transition Zone (CATZ) is not a diffuse or transient feature but a persistent, tectonically significant structure accommodating left-lateral shear between the oppositely moving eastern and western sectors of the Anatolian Plate.

The spatial correlation between LiCSAR-derived shear patterns, GNSS velocity gradients, and stress-field rotations defines the CATZ as a crustal- to lithospheric-scale transition zone linking the North and East Anatolian fault systems to the subduction domains of the Hellenic and Cyprus arcs. Deformation rates of approximately 4 mm/yr and systematic transitions from strike-slip to transtensional and compressional regimes reflect the mechanical response of the Anatolian lithosphere to simultaneous plate convergence, slab rollback, and internal crustal weakening.

Stress inversion results delineate four distinct deformation subzones along the CATZ. The northern zones (1–2) show NW–SE compressional stresses associated with the East Anatolian regime, while Zone 3 exhibits NW–SE extension, marking the shift toward central Anatolia. The southernmost Zone 4 reflects complex deformation due to Mediterranean subduction processes, with NNE–SSW compression and localized transtension influenced by the Hellenic arc. This pattern confirms that the CATZ is both a mechanical and tectonic transition zone.

Collectively, geodetic and seismic observations suggest that the CATZ reflects the interaction of three complementary geodynamic processes: a lithospheric slab tear or detachment, Moho depth–related crustal heterogeneity, and a localized crustal thickness transition forming a rheological boundary. Together, these mechanisms promote distributed strain localization across the CATZ and have sustained long-term activity since the late Miocene, linking upper-crustal deformation with deeper lithospheric reorganization.

From a broader perspective, this study demonstrates the value of integrating satellite geodesy and geophysical imaging to resolve diffuse continental deformation. The recognition of the CATZ as a fundamental lithospheric transition zone revises the conventional view of Anatolia as a rigidly translating microplate and highlights the critical role of internal deformation in accommodating plate motions. Continued integration of InSAR, seismic tomography, and gravity modeling will further refine understanding of the deep structure and evolving dynamics of this key tectonic domain.

## Figures and Tables

**Table 1:** Parameters of stress inversion of the transition zone from northern to southern in Central Anatolia.

Zone	Data	Used Data	σ1	σ2	σ3	R’	Tectonic Regime	SH_max_	St. Dev.	α	QRfm
All	86	86	52/334	37/171	08/075	0.77	TT	164	7.5	35	C
Zone 1	16	16	01/340	88/102	02/250	1.55	PS	159	9.3	20.3	C
Zone 2	36	36	01/340	86/237	04/070	1.53	PS	160	12.5	12.5	B
Zone 3	22	22	83/172	06/023	04/293	0.43	PE	022	19.5	13.9	B
Zone 4	14	14	65/236	02/331	25/062	0.90	TT	152	7.4	43	C

Zone number, number of data points in each zone, number of data points used in stress tensor solution; reduced stress tensor parameters: orientation (plunge/strike) of the principal stress axes (σ1, σ2, and σ3) and stress regime index (R’); misfit angle (α), and quality rank for the stress inversion of focal mechanisms (QRfm) as defined in the World Stress Map. TT: Transtensional, PS: Pure Strike-Slip, PE: Pure Extensional.

## Data Availability

The data presented in this study are available on request from the corresponding author.

## Supplementary Materials

The following supporting information can be downloaded at: https://www.mdpi.com/article/10.3390/s26020505/s1, Table S1: Database of seismic events used in this study, organized by zones along the Central Anatolian Transition Zone (CATZ). The dataset was compiled from the AFAD earthquake catalog (2024).
