# Supplementary material for: Delineating the Central Anatolia Transition Zone (CATZ): Constraints from Integrated Geodetic (GNSS/InSAR) and Seismic Data
Source: Sensors (Basel). 2026 Jan 12;26(2):505. doi: 10.3390/s26020505 (PMC12846039; doi:10.3390/s26020505)
Supplement: Supplementary file 1 [file sensors-26-00505-s001.zip › sensors-4027515-supplementary.pdf]

No	Date	Latitude	Longitude	Depth	Magnitude	Strike 1	Dip 1	Rake 1	SHmax	SHmin	Regime Index	Regime Code	Source
**Zone1**	07/11/1999	40.57	31.36	15.00	4.9	47	25	51	158	068	2.5	TF	GCMT
**Zone1**	12/11/1999	40.93	31.25	18.00	7.1	268	54	-167	129	039	1.0	NS	GCMT
**Zone1**	16/08/2010	40.84	31.63	10.83	4.0	97	80	160	144	054	1.5	SS	AFAD
**Zone1**	24/07/2013	41.71	32.39	20.09	4.0	330	43	-127	173	083	0.5	NF	AFAD
**Zone1**	24/11/2013	40.76	31.87	19.70	4.9	64	37	76	163	073	2.5	TF	AFAD
**Zone1**	04/09/2014	41.61	32.48	16.85	4.1	80	45	124	148	058	2.5	TF	AFAD
**Zone1**	07/04/2018	40.85	31.63	10.47	4.7	21	82	-3	156	066	1.5	SS	AFAD
**Zone1**	03/05/2018	40.84	31.65	9.45	4.0	187	61	-17	146	056	1.5	SS	AFAD
**Zone2**	06/06/2000	40.75	32.70	15.00	6.0	356	39	-47	151	061	0.5	NF	GCMT
**Zone2**	30/07/2005	39.46	33.10	14.4	5.2	214	87	-2	169	079	1.5	SS	GCMT
**Zone2**	20/12/2007	39.43	33.10	12.00	5.7	214	73	17	167	077	1.5	SS	GCMT
**Zone2**	26/12/2007	39.55	33.05	15.00	5.6	231	67	5	005	095	1.5	SS	GCMT
**Zone2**	31/01/2008	40.30	33.25	17.10	4.9	193	77	13	147	057	1.5	SS	GCMT
**Zone2**	19/02/2010	39.96	33.15	17.47	4.0	200	79	-31	158	068	1.5	SS	AFAD
**Zone2**	19/10/2010	39.39	33.11	7.02	4.3	141	37	166	012	102	2.0	TS	AFAD
**Zone2**	22/12/2010	40.21	33.27	19.15	3.8	303	85	176	168	078	1.5	SS	AFAD
**Zone2**	02/08/2016	40.31	33.36	12.23	4.2	30	87	1	165	075	1.5	SS	AFAD
**Zone2**	12/02/2019	40.55	33.00	17.66	4.7	102	69	-172	146	056	1.5	SS	AFAD
**Zone2**	14/09/2019	40.73	32.96	7.96	4.7	285	87	-179	150	060	1.5	SS	AFAD
**Zone2**	14/09/2019	40.72	32.94	5.83	4.8	286	86	-176	151	061	1.5	SS	AFAD
**Zone2**	14/09/2019	40.73	32.96	8.41	4.3	282	82	-167	146	056	1.5	SS	AFAD
**Zone2**	14/09/2019	40.72	32.98	10.22	4.1	285	86	-174	150	060	1.5	SS	AFAD
**Zone2**	14/09/2019	40.73	32.98	10.61	4.3	286	86	-178	151	061	1.5	SS	AFAD
**Zone2**	23/01/2020	40.11	33.26	13.65	4.5	290	89	-176	155	065	1.5	SS	AFAD
**Zone2**	23/06/2020	40.73	32.97	8.77	4.2	279	76	175	145	055	1.5	SS	AFAD
**Zone2**	10/01/2021	40.04	33.32	13.19	4.5	116	82	160	163	073	1.5	SS	AFAD
**Zone3**	15/12/2000	38.40	31.35	15.00	6.0	285	41	-100	111	021	0.5	NF	GCMT
**Zone3**	03/02/2002	38.62	31.21	15.00	6.5	269	39	-71	078	168	0.5	NF	GCMT
**Zone3**	03/02/2002	38.52	31.22	15.00	5.3	76	43	-70	063	153	0.5	NF	GCMT
**Zone3**	10/09/2009	37.76	32.51	21.70	4.8	28	42	-82	022	112	0.5	NF	GCMT
**Zone3**	11/09/2009	37.72	32.50	18.80	4.9	26	39	-76	017	107	0.5	NF	GCMT
**Zone3**	27/07/2011	38.32	31.88	17.79	4.8	127	23	-82	123	033	0.5	NF	AFAD
**Zone3**	03/04/2017	38.48	31.79	13.84	4.0	201	58	-40	168	078	1.0	NS	AFAD
**Zone3**	09/02/2021	38.59	31.63	7.01	4.7	337	72	-143	015	105	1.0	NS	AFAD
**Zone3**	09/02/2021	38.59	31.64	4.61	4.1	208	49	-108	040	130	0.5	NF	AFAD
**Zone3**	08/11/2021	37.86	32.11	6.82	5.1	206	48	-87	024	114	0.5	NF	AFAD
**Zone3**	31/01/2022	37.87	32.11	6.99	4.2	215	47	-77	026	116	0.5	NF	AFAD
**Zone4**	01/06/1977	35.79	31.48	58.6	5.6	132	64	155	003	093	1.5	SS	GCMT
**Zone4**	15/12/2011	37.00	30.29	77.6	4.3	101	87	157	147	057	1.5	SS	AFAD
**Zone4**	27/06/2013	36.75	31.23	50.61	4.1	116	63	-12	074	164	1.5	SS	AFAD
**Zone4**	08/12/2013	36.69	31.25	65.00	5.0	325	82	-15	101	011	1.5	SS	AFAD
**Zone4**	25/12/2013	36.98	31.04	52.38	4.1	249	61	-2	024	114	1.5	SS	AFAD
**Zone4**	12/09/2018	36.05	31.21	36.68	5.2	296	69	-151	155	065	1.5	SS	AFAD
**Zone4**	26/10/2018	36.08	31.22	52.85	4.1	95	75	168	141	051	1.5	SS	AFAD
